# No Bureau of Vital Statistics for Texas

**Published:** 1903-04

**Authors:** 


### NO BUREAU OF VITAL STATISTICS FOR TEXAS.

IT HAS LONG BEEN A REPROACH to Texas that she takes no account of her population, though she does of her pigs; and that there is no record from which it could be shown in court that any decedent had ever been born, or if so, that he was legitimate, or that he had ever died. It has been a matter of surprise and disappointment to the State Association of Physicians that all their efforts in the past to secure legislaton to provide this very necessary measure should have failed.

The registration of the life statistics of a people is fundamental; it is the starting point; the base upon which sanitary science is founded. Without a knowledge of the number of deaths and the causes thereof, and the geographical distribution of diseases, no measures of prevention can ever be intelligently instituted, and no sanitary reform or progress is possible. It is therefore a matter of regret and mortification that we announce that we were not able, in the face of much and unexpected opposition, obstruction, and provoking delays, to secure the final passage through both houses of the late Legislature of a good law creating a bureau for the record and preservation of the vital statistics of Texas. The bill as passed is not what the committee asked for, nor what is needed. It was emasculated, and is not worth a cent. For instance, the original bill provided for the appointment of a registrar, who should be a sanitarium and familiar with statistics as the head of the bureau or sub-department. The work can not be done by clerks. The Senate committee cut the registrar out and made no provision for the employment of one. Again, to placate a member who could see the matter only from a personal standpoint, it was necessary to the final passage of the act to cut out

"illegitimate or legitimate," because he said it would be "a shame to expose a girl who had been victimized." The United States Government is making an effort to get the States to adopt a uniform registration system. Texas should fall in line, and the "Red Back" regrets that the opportunity to do so was not improved by the Legislature.

The Vital Statistics bill appears elsewhere.

---

Before leaving the subject of the Vital Statistics law, I want to say to my readers, and especially to the medical profession and the people of Texas, that Texas owes a heavy debt of gratitude to the Honorable Alf. F. Harper, Senator from Limestone, for the intelligence and zeal with which he took hold of, and the personal interest he manifested in the bill and the Railway Sanitation bill, his hearty co-operation with the Association's committee, and his final success in getting them through the Senate. I don't want any Texan ever to forget Judge Harper, but if he ever wants anything at their hands I want him to have it. In the House the bills were ably and skillfully handled, and finally put through by the Honorable (Dr.) John M. Johnson, Representative from Giddings, Lee County (the author of the Railway Sanitation bill, which he and his lieutenants in the House, and Senator Harper in the Senate, put through so successfully). Dr. Johnson was supported and greatly assisted by Judge Nicholson of Laredo, Judge Knight of Bosque, and Honorable W. J. Bryan of Abilene. The State Medical Association's committee are greatly indebted to these gentlemen, and I publicly take off my hat to them.

